# Age-Structured Clinical Background Is More Strongly Associated with C-Reactive Protein Levels than Individual Respiratory Viruses During Respiratory Virus Testing

**DOI:** 10.3390/pathogens15060583

**Published:** 2026-05-28

**Authors:** Sung Hun Jang, Bo Kyeung Jung, Jae-Sik Jeon, Jeong Su Han, Jae Kyung Kim

**Affiliations:** 1 Department of Medical Laser, Graduate School of Medicine, Dankook University, Cheonan-si 31116, Republic of Korea; well8143@naver.com; 2 Department of Laboratory Medicine, College of Medicine, Dankook University, Cheonan-si 31116, Republic of Korea; lovegodmother@hanmail.net; 3 Department of Biomedical Laboratory Science, College of Health Sciences, Dankook University, Cheonan-si 31116, Republic of Korea; zenty87@naver.com (J.-S.J.); jshan1162@naver.com (J.S.H.)

**Keywords:** age stratification, C-reactive protein, diagnostic interpretation, multiplex PCR, respiratory viruses

## Abstract

We aimed to characterize age-stratified C-reactive protein (CRP) patterns across respiratory virus infections, assess age-related CRP shifts in virus-not-detected-by-PCR episodes, and evaluate the independent associations of age and virus type with CRP levels. We retrospectively analyzed 19,002 test-level episodes with paired respiratory PCR and serum CRP results from a single tertiary-care center between 2008 and 2024. Episodes were classified as virus-not-detected-by-PCR or virus-positive according to multiplex PCR results; the former was not considered a healthy control and may include off-panel infections, bacterial/mixed infections, false-negative results, or non-infectious inflammation. Descriptive analyses and multivariable linear regression of log-transformed CRP were used to assess adjusted associations. Median CRP increased with age in both virus-positive and virus-not-detected-by-PCR episodes, rising from 0.38 to 7.42 mg/dL across age groups in the latter. Age showed the strongest association with CRP. Adenovirus showed a positive adjusted association, whereas influenza A/B, respiratory syncytial virus A/B, and parainfluenza virus types 1 and 3 showed selective negative associations. Overall, CRP variation was more strongly associated with age-related clinical background than with virus type, although selective virus-associated differences were observed, supporting the interpretation of CRP as a non-specific, composite host-response indicator within broader clinical contexts.

## 1. Introduction

Respiratory virus infections are among the most frequent causes of outpatient visits and emergency department presentations, encompassing a broad clinical spectrum from mild upper respiratory illness to severe pneumonia and respiratory failure [1]. In early clinical encounters, differentiating viral from bacterial infection based on symptoms and physical findings alone is unreliable, often leading to empirical antibiotic prescription [2]. This diagnostic uncertainty complicates early clinical decision-making and may contribute to inappropriate or unnecessary antibiotic use [3].

C-reactive protein (CRP) is widely used as a rapid inflammatory biomarker in patients with suspected respiratory infection, but it is not pathogen-specific and cannot reliably distinguish viral from bacterial infection [4,5]. CRP responses may also differ by viral species; for example, adenovirus can be associated with relatively strong inflammatory responses, whereas respiratory syncytial virus (RSV) and several parainfluenza viruses have been associated with low or modest CRP elevations [6,7]. However, detailed, age-stratified, virus-specific CRP distributions remain limited, complicating determining whether observed CRP differences primarily reflect viral identity or the age-related clinical background in which viral detection occurs.

Patient age further complicates CRP interpretation because developmental and aging-related immune changes can alter baseline inflammatory status and host responses to infection [8,9,10]. Population-based studies have also shown age- and sex-related variation in CRP distributions, indicating that identical CRP values may have different clinical implications in pediatric, adult, and older-adult patients [11]. Therefore, virus-associated CRP values should be interpreted against the patient’s age-related clinical background rather than by viral detection status alone.

Although multiplex respiratory PCR testing is widely available and enables rapid identification of viral pathogens, the detection of a virus alone has not consistently translated into reduced antibiotic prescribing in routine clinical practice [12]. This gap highlights the need for diagnostic stewardship approaches that integrate pathogen detection with host-response indicators in a clinically interpretable framework. Therefore, understanding the age-stratified virus-associated CRP distributions and age-dependent CRP distribution observed in virus-not-detected-by-PCR episodes may provide essential context for more accurate interpretation of CRP values and support more rational antibiotic use. In particular, age-related CRP elevation in virus-not-detected-by-PCR episodes may represent an important clinical background against which virus-associated CRP patterns should be interpreted.

In this study, we aimed to describe age-stratified, virus-specific CRP profiles using large-scale and real-world data from a single tertiary-care center. We also sought to provide a quantitative clinical context for interpreting CRP during respiratory virus testing. Specifically, we examined whether CRP variation was closely associated with pathogen identity or with the age-related clinical background in which viral detection occurred. To address this question, we evaluated age-stratified virus-specific CRP patterns and characterized age-dependent CRP distributions in virus-not-detected-by-PCR episodes. These virus-not-detected episodes were interpreted as a clinically relevant background context rather than as a healthy reference group. We then examined age-group and virus-specific associations in adjusted models. Through this approach, we aimed to clarify how age reshapes virus-associated CRP values and to support age- and pathogen-aware interpretations of CRP in routine clinical practice.

## 2. Materials and Methods

### 2.1. Study Design, Population, and Study Period

This retrospective observational study was conducted at Dankook University Hospital, a tertiary-care hospital in Cheonan-si, Republic of Korea. The study period spanned from October 2008 to December 2024. Eligible episodes were defined as clinical encounters in which patients with respiratory symptoms, including cough, sputum production, or dyspnea, underwent multiplex respiratory virus PCR testing and serum CRP measurement during the same encounter. Paired PCR and CRP results were linked using the hospital information system. The unit of analysis was a test-level episode. Repeated tests from the same patient were retained as separate episodes in the main analysis, and their potential influence was assessed in a sensitivity analysis restricted to the first eligible episode per patient. In total, 19,002 test episodes were included.

### 2.2. Specimen Handling and Respiratory Virus Detection

Nasopharyngeal swab specimens were collected for respiratory virus testing. Specimens were processed immediately or stored at 4 °C and analyzed within 24 h. Viral nucleic acids were extracted using the QIAamp Viral RNA Mini Kit (QIAGEN, Hilden, Germany) according to the manufacturer’s instructions.

During the study period, multiplex PCR platforms were operated in accordance with laboratory standard operating procedures:

- 2008–2012: Seeplex Respiratory Virus (RV) series (Seegene, Seoul, Republic of Korea) with gel electrophoresis-based detection.
- 2013–2024: AdvanSure RV and RV-Plus real-time RT-PCR assays (LG Chem, Seoul, Republic of Korea) conducted on the SLAN real-time PCR system.

To improve longitudinal comparability despite the platform change during the study period, analyses were restricted to respiratory viruses that were consistently targeted across both assay periods. These included adenovirus, influenza A/B, respiratory syncytial virus A/B (RSV A/B), rhinovirus, enterovirus, parainfluenza virus types 1–3, human metapneumovirus (hMPV), and bocavirus. However, seasonal human coronaviruses (e.g., 229E, OC43, and NL63), which were not uniformly targeted across platforms, were excluded from the analysis.

### 2.3. CRP Measurement

CRP levels were quantified by automated immunoturbidimetry using the Cobas 8000 modular analyzer with Tina-quant C-Reactive Protein Gen.3 reagent (Roche Diagnostics, Mannheim, Germany). Although the manufacturer reports the analytical range in mg/L, CRP values were recorded in mg/dL in the laboratory information system (LIS); accordingly, all analyses were performed using mg/dL. The reportable range was 0.03–70 mg/dL, with direct measurement > 0.03–35 mg/dL and automatic 1:2 dilution for values > 35 mg/dL, extending the upper limit to 70 mg/dL. Final LIS-reported values were analyzed without further adjustment or substitution. Blood samples were centrifuged after clotting, serum was stored at 2–8 °C, and assays were performed within 24 h. Internal and external quality-control procedures were maintained throughout the study period.

### 2.4. Data Integration, Case Definitions, and Covariates

Patient age at testing was calculated from the date of birth and expressed in years. To evaluate age-related differences in host inflammatory responses, episodes were categorized into five age groups: <1 year, 1–12 years, 13–18 years, 19–64 years, and ≥65 years. Episodes in which no respiratory virus target was detected by multiplex PCR were classified as virus-not-detected-by-PCR episodes, whereas episodes with at least one detected respiratory virus target were classified as virus-positive episodes. Respiratory virus-positive episodes included both single-virus detections and coinfections; therefore, episodes with multiple detected viruses were included in the analytic dataset rather than excluded. This approach was chosen because multiple-virus detections are not uncommon in clinical multiplex respiratory PCR testing, particularly in pediatric populations, and excluding these episodes could reduce the representativeness of the real-world analytic cohort. Owing to the interpretive difficulty of attributing CRP variation to a specific virus in episodes with multiple viral detections, an additional analysis was performed using a strict single-virus-positive subset. This subset was defined as episodes with exactly one detected viral target among the study viruses. For descriptive virus-specific summaries, virus-positive episodes were expanded into a long-format dataset so that one coinfected episode could contribute to more than one virus-specific category. In contrast, for multivariable regression, the original episode-level dataset was retained, and coinfection was accounted for by including both the total number of detected viruses per episode and virus-specific binary indicators in the same model. Virus-not-detected-by-PCR episodes were analyzed separately as a virus-not-detected-by-PCR clinical background rather than a healthy reference group. This category may have included episodes with pathogens not covered by the PCR panel or false-negative results. Therefore, it was interpreted as a clinical background group rather than a definitive true-negative group. Covariates included age group, sex, calendar month, and calendar year. Multivariable regression models additionally incorporated the total number of detected viruses per episode and virus-specific binary indicators. Data on bacterial co-infection, antibiotic exposure at the time of testing, specific comorbidities (such as chronic obstructive pulmonary disease), autoimmune disease, malignancies, immunosuppressive status, and symptom duration were not uniformly available in the retrospective laboratory dataset. Therefore, these variables could not be incorporated as covariates in the multivariable models or used to define a reliable immunocompetent subgroup without chronic inflammatory conditions.

Episodes with missing age, sex, PCR, or CRP data would have been excluded; however, no eligible episodes had missing values for these variables after data integration.

### 2.5. Statistical Analysis

As CRP values showed a right-skewed distribution, unadjusted descriptive comparisons between groups were performed using non-parametric methods, including the Kruskal–Wallis test. In Table 1, *p*-values from Kruskal–Wallis tests were retained for transparency; however, virus rows containing one or more age–virus cells with fewer than 10 observations were indicated with a dagger symbol (†) to denote that these *p*-values and subgroup medians should be interpreted as descriptive only rather than as robust inferential comparisons. 

Descriptive analyses were used to characterize virus-specific CRP distributions, and regression analyses were used to estimate adjusted episode-level associations. For virus-specific descriptive analyses, the data were restructured into a virus-positive long-format dataset that included coinfected episodes. For adjusted analyses, multivariable linear regression models were fitted in the full episode-level cohort, including coinfected episodes, using log-transformed CRP values [log(CRP + 1)] as the dependent variable. This transformation was applied to reduce right skewness and stabilize variance, thereby enabling estimation of adjusted associations. No CRP values were recorded as 0 mg/dL in the analytic dataset. The +1 offset was used to provide a stable transformation of very low CRP values. Because the dependent variable was log(CRP + 1), back-transformed coefficients were interpreted as approximate relative differences from the transformed model rather than exact proportional changes in raw CRP concentrations. Independent variables included age group, sex, calendar month, calendar year, the total number of viruses detected per episode, and binary indicators representing the presence or absence of each virus. Inclusion of calendar year was intended, in part, to account for long-term temporal variation across the study period, including potential shifts associated with changes in diagnostic platforms and testing practices. In the regression models, the reference categories were age < 1 year for age-group comparisons, female sex for sex comparisons, January for calendar month, and 2008 for calendar year. Age group and sex were entered as separate categorical predictors without an interaction term; therefore, this coding was used to estimate the age gradient from the youngest group and the sex difference separately, rather than to define females aged <1 year as a clinically meaningful combined reference group. 

As primary sensitivity analyses, the multivariable linear regression model was refitted in several restricted datasets. First, to assess the potential influence of repeated testing within individuals, a sensitivity analysis was performed using a patient-level dataset restricted to the first eligible episode per patient. The same multivariable linear regression model was then refitted, and the direction, magnitude, and statistical significance of the associations were compared with those of the main analysis. Second, a strict single-virus-positive subset was analyzed to evaluate virus-specific associations after excluding episodes with multiple viral detections, thereby reducing attributional ambiguity. In the strict single-virus-positive subset analysis, virus-specific indicators were perfectly collinear because each episode contained exactly one detected viral target; therefore, bocavirus was specified as the omitted reference category to avoid multicollinearity. Third, episodes with very high CRP concentrations (>20 mg/dL) were excluded, and the same multivariable model used in the main analysis was refitted to assess whether the observed associations were driven by a small number of markedly inflamed episodes. The same adjustment covariates were retained in these sensitivity analyses, as applicable. Fourth, to assess whether the main findings were robust to platform-related variation, an additional sensitivity analysis was performed after restricting the dataset to episodes tested during the later real-time RT-PCR platform period from 2013 to 2024. The same multivariable linear regression model was refitted in this restricted dataset, and the direction and magnitude of age- and virus-specific associations were compared with those of the main analysis. Virus-not-detected-by-PCR episodes were analyzed separately to describe age-stratified CRP distributions in episodes without detected respiratory virus targets.

All statistical analyses were two-sided, and *p* < 0.05 was considered statistically significant. Analyses were performed using R software (R Foundation for Statistical Computing, version 4.3.3, Vienna, Austria).

### 2.6. Ethical Considerations

This retrospective study involved anonymized existing data and was conducted in accordance with the ethical principles of the Declaration of Helsinki of 1975, as revised in 2013. The study protocol, including the use, extraction, and retrospective analysis of existing data, was approved by the Institutional Review Board of Dankook University Hospital on 5 March 2026 (IRB approval no. DKUH IRB 2026-01-013-001), and the requirement for informed consent was waived. Data were retrospectively extracted on 7 March 2026 following IRB approval.

No intervention was performed, no additional specimens were collected, and there was no direct patient contact or involvement for the purpose of this research.

## 3. Results

### 3.1. Age-Stratified Virus-Specific CRP Distributions

In total, 19,002 episodes were included in the analysis. The median age was 3.0 years, with an interquartile range of 0–44 years and an overall age range of 0–98 years. The age-group distribution of the episodes was as follows: 5506 from those aged <1 year, 7407 from those aged 1–12 years, 547 from those aged 13–18 years, 2288 from those aged 19–64 years, and 3254 from those aged ≥65 years. Among the detected viruses, rhinovirus was the most frequent, with 3559 virus-positive observations, followed by adenovirus (*n* = 1834), RSV A (*n* = 1460), RSV B (*n* = 1258), influenza A (*n* = 962), parainfluenza virus 3 (*n* = 904), and hMPV (*n* = 802) (Table 1).

**Figure 1:**
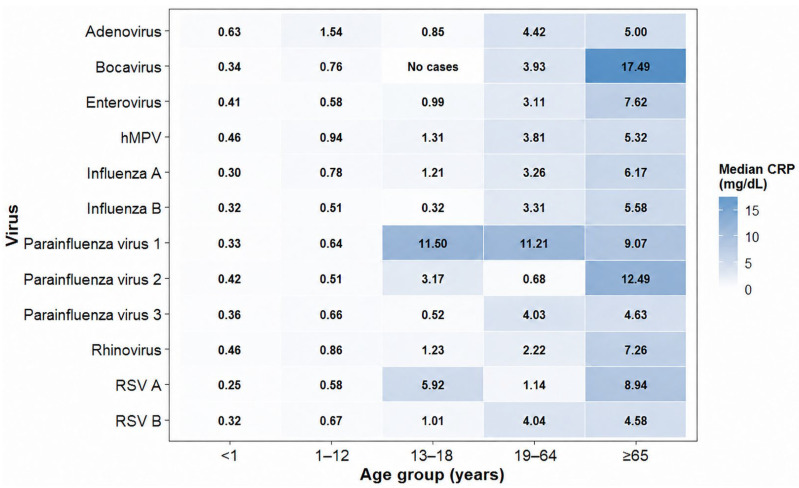
Heatmap of age-stratified median CRP levels across respiratory viruses. Cells show the median CRP concentration (mg/dL) for each virus-positive group across five age strata (<1, 1–12, 13–18, 19–64, and ≥65 years). Darker shading indicates higher median CRP values. The number of observations contributing to each virus–age cell is provided in Table S5. Estimates for very small age–virus subgroups should be regarded as descriptive rather than inferential, particularly when fewer than 10 observations were available in a given cell. hMPV, human metapneumovirus; RSV, respiratory syncytial virus; CRP, C-reactive protein.

Comparison of the median CRP values and interquartile ranges across age groups showed an overall upward shift in the CRP distribution with increasing age for several major viruses (Figure 1). This pattern was particularly pronounced for adenovirus, influenza A, rhinovirus, hMPV, RSV A, and RSV B. Within-virus comparisons indicated age-related differences in CRP distributions for several frequently detected viruses. For virus rows containing one or more age-specific cells with fewer than 10 observations, *p*-values were retained in Table 1 for transparency and indicated with a dagger symbol (†) to denote that these comparisons should only be interpreted as descriptive because of limited subgroup stability. The number of observations contributing to each virus–age cell is additionally summarized in Table S5 to facilitate assessment of the stability of the descriptive median estimates.

### 3.2. Age-Dependent CRP Distribution in Virus-Not-Detected-by-PCR Episodes

**Figure 2:**
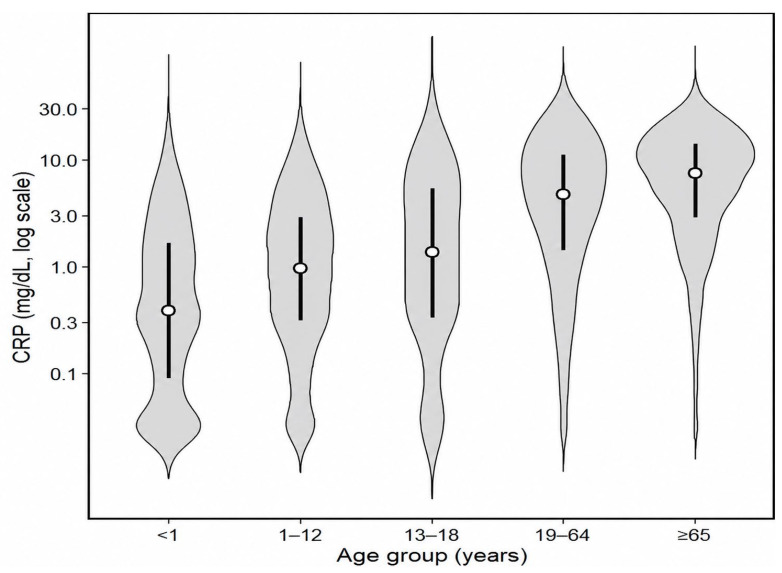
Age-dependent upward shift in CRP distributions among virus-not-detected-by-PCR episodes. Violin plots depict CRP concentration distributions across age groups among virus-not-detected-by-PCR episodes. White circles indicate the median, black vertical lines represent the interquartile range, and violin width reflects the relative density of observations at each CRP level. The y-axis is presented on a logarithmic scale. CRP, C-reactive protein.

Among virus-not-detected-by-PCR episodes, CRP concentrations significantly differed across the five age groups (*p* < 0.001; Table 2). The median CRP level progressively increased with age, from 0.38 mg/dL (IQR, 0.09–1.62) in those aged <1 year to 0.95 mg/dL (IQR, 0.31–2.88) in those aged 1–12 years, 1.36 mg/dL (IQR, 0.33–5.33) in those aged 13–18 years, 4.71 mg/dL (IQR, 1.42–10.96) in those aged 19–64 years, and 7.42 mg/dL (IQR, 2.87–13.83) in those aged ≥65 years (Figure 2).

### 3.3. Multivariable Analysis of Factors Associated with CRP

To evaluate the independent associations of age group and virus type with CRP levels, we performed a multivariable linear regression analysis using log-transformed CRP values [log(CRP + 1)] as the dependent variable (Table S1). After simultaneous adjustment for sex, calendar month, calendar year, total number of detected viruses, and the presence of other viruses, CRP remained strongly and independently associated with age. Using age < 1 year as the reference category for age-group comparisons, the model-based estimates showed progressively higher transformed CRP levels across age groups, corresponding to approximate back-transformed relative differences of 28.2% in those aged 1–12 years, 57.0% in those aged 13–18 years, 189.7% in those aged 19–64 years, and 291.9% in those aged ≥65 years (all *p* < 0.001). To aid interpretation on the original clinical scale, this adjusted age gradient was interpreted alongside the age-stratified untransformed CRP medians observed in virus-not-detected-by-PCR episodes, which increased from 0.38 mg/dL in those aged <1 year to 0.95 mg/dL in those aged 1–12 years, 1.36 mg/dL in those aged 13–18 years, 4.71 mg/dL in those aged 19–64 years, and 7.42 mg/dL in those aged ≥65 years. Male sex showed an approximate positive back-transformed relative difference of 6.3% compared with female sex (*p* < 0.001). In contrast, the total number of detected viruses was not significantly associated with CRP after adjustment (*p* = 0.142), and calendar month was not strongly associated with CRP overall, although a small negative association was observed for July compared with January.

**Figure 3:**
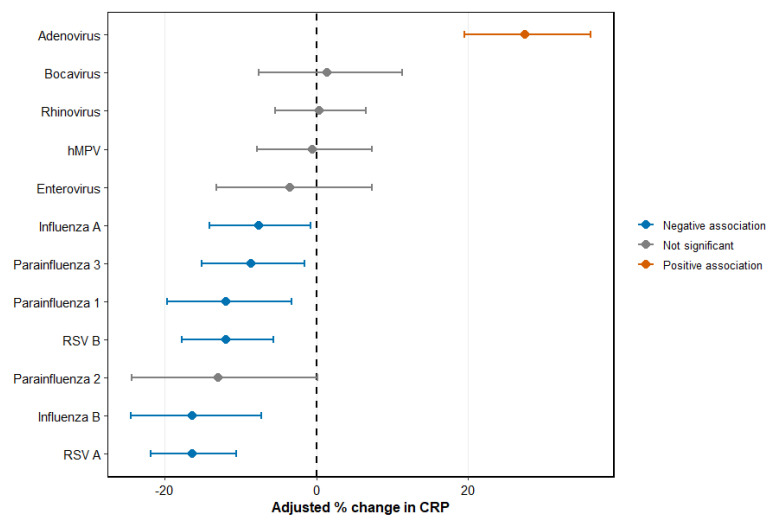
Adjusted associations between respiratory viruses and serum CRP levels. Forest plot showing the adjusted percent change in CRP associated with each respiratory virus. Dots indicate point estimates, and horizontal bars represent 95% confidence intervals. The vertical dashed line at 0 indicates no association. Estimates to the left of 0 indicate relatively low adjusted CRP levels within the multivariable model, as opposed to suppression of CRP below a healthy or pre-infection baseline. These estimates should be interpreted as relative differences among clinically tested episodes after adjustment for age group, sex, calendar month, calendar year, total number of detected viruses, and other virus indicators. Blue indicates a significant negative association, orange indicates a significant positive association, and gray indicates no statistically significant association. CRP, C-reactive protein; RSV, respiratory syncytial virus.

With respect to individual viruses, adenovirus showed an approximate positive back-transformed relative difference of 27.5% (*p* < 0.001), whereas influenza A (−7.6%, *p* = 0.031), influenza B (−16.3%, *p* < 0.001), RSV A (−16.4%, *p* < 0.001), RSV B (−11.9%, *p* < 0.001), parainfluenza virus type 1 (−11.9%, *p* = 0.008), and parainfluenza virus type 3 (−8.6%, *p* = 0.018) showed approximate negative back-transformed relative differences within the multivariable framework. Parainfluenza virus type 2 also showed an approximate negative back-transformed relative difference, although not significant (−13.0%, *p* = 0.052). By contrast, hMPV, rhinovirus, enterovirus, and bocavirus showed no significant independent associations with transformed CRP levels after adjustment (Figure 3).

In sensitivity analyses restricted to the first eligible episode per patient, the overall pattern of associations was largely preserved (Table S2). Age remained the strongest independent correlate of CRP, male sex retained a modest positive association, and adenovirus remained positively associated with CRP. In contrast, influenza A/B, RSV A/B, and parainfluenza virus types 1 and 3 continued to show inverse associations, whereas hMPV, rhinovirus, enterovirus, and bocavirus remained not significantly associated with CRP.

To address the difficulty of attributing CRP variation to a specific virus in episodes with multiple viral detections, we additionally performed a multivariable linear regression analysis restricted to a strict single-virus-positive subset (*n* = 7389) (Table S3). In this restricted analysis, the overall interpretation was preserved: age remained the factor most strongly associated with CRP, with approximate back-transformed relative differences of 28.0% in those aged 1–12 years, 48.0% in those aged 13–18 years, 180.0% in those aged 19–64 years, and 289.0% in those aged ≥65 years (all *p* < 0.001), whereas male sex was not significantly associated with CRP. With respect to individual viruses, adenovirus remained positively associated with CRP (+31.1%, *p* < 0.001), whereas influenza B (−18.4%, *p* = 0.009), RSV A (−19.1%, *p* = 0.002), RSV B (−15.5%, *p* = 0.014), parainfluenza virus type 1 (−17.5%, *p* = 0.011), and parainfluenza virus type 3 (−15.1%, *p* = 0.017) remained negatively associated. Influenza A, hMPV, parainfluenza virus type 2, rhinovirus, and enterovirus were not significantly associated with CRP in this restricted model. As a second primary sensitivity analysis, episodes with very high CRP concentrations (>20 mg/dL) were excluded to evaluate whether the main findings were driven by a small number of markedly inflamed episodes (Table S4). A total of 646 episodes (3.4%) were excluded, leaving 18,356 episodes in the restricted dataset. The overall pattern remained consistent with the main analysis. Age remained the strongest independent correlate of CRP, with approximate back-transformed relative differences of 27.4% in those aged 1–12 years, 49.8% in those aged 13–18 years, 147.0% in those aged 19–64 years, and 234.0% in those aged ≥65 years, compared with those aged <1 year. Adenovirus retained a positive association with CRP (+30.7%, *p* < 0.001), whereas influenza B (−14.2%, *p* = 0.002), RSV A (−14.0%, *p* < 0.001), RSV B (−9.4%, *p* = 0.003), and parainfluenza virus type 1 (−11.2%, *p* = 0.008) retained negative associations.

To further assess the potential influence of platform-related variation, an additional sensitivity analysis was performed after restricting the dataset to the later real-time RT-PCR platform period from 2013 to 2024 (*n* = 13,063; Table S6). The dominant age-related pattern was preserved, with adjusted CRP levels remaining progressively higher across age groups. Age-associated relative differences were 25.4% in those aged 1–12 years, 53.2% in those aged 13–18 years, 180.3% in those aged 19–64 years, and 281.4% in those aged ≥65 years compared with those aged <1 year. Adenovirus retained a positive association with CRP (+26.1%, *p* < 0.001), whereas influenza A/B, RSV A/B, and parainfluenza virus types 1 and 3 retained negative associations. The total number of positive viral targets was not significantly associated with CRP (*p* = 0.195).

## 4. Discussion

In this large single-center retrospective study, the principal pattern of CRP variation in respiratory virus testing was an age-related upward shift observed across multiple virus-positive groups, with a similar pattern also seen in virus-not-detected-by-PCR episodes. Notably, virus-not-detected-by-PCR episodes should not be interpreted as a healthy baseline or control group. Rather, these cases represent a clinically heterogeneous background group in which PCR panel-targeted respiratory viruses were not detected. This category may include infections caused by pathogens not covered by the PCR panel, false-negative PCR results related to specimen timing or assay performance, bacterial or mixed infections, and non-infectious inflammatory conditions [13,14,15]. In adjusted analyses, age showed the strongest association with CRP, whereas independent virus-specific associations were selective. Collectively, these findings suggest that, in respiratory virus testing, CRP is more strongly patterned by an age-structured clinical background than by viral identity alone, with selective pathogen-specific differences superimposed. Accordingly, CRP interpretation should not rely on viral detection alone but should be contextualized within the patient’s age-related clinical background and the non-specific nature of CRP as a host-response marker. One of the most clinically relevant observations, as also reported in previous studies, was that CRP increased progressively across age groups [16,17]. In the present study, this pattern was also clearly evident in virus-not-detected-by-PCR episodes. Median CRP values in virus-not-detected-by-PCR adults, particularly older adults, were substantially higher than those observed in younger age groups and some pediatric virus-positive strata. This finding suggests that identical CRP values may represent fundamentally different biological and clinical contexts depending on the patient’s position within the age-specific background distribution.

Several mechanisms may contribute to this age-related upward shift in CRP distributions. Aging is associated with chronic low-grade systemic inflammation (“inflammaging”), characterized by persistent elevation of pro-inflammatory cytokines, such as interleukin-6, which directly stimulates hepatic CRP production [18,19,20]. Additionally, adults, particularly those presenting to tertiary-care settings, are more likely to have comorbid conditions, such as cardiovascular disease, chronic lung disease, or metabolic disorders, all of which may contribute to higher age- and comorbidity-related CRP distributions [21,22,23]. Consequently, CRP elevation in adults may reflect a combination of acute infection, chronic clinical background associated with aging and comorbidity, and case-mix severity rather than pathogen-specific effects alone.

From a clinical perspective, these findings do not negate the clinical utility of established CRP cut-offs but suggest that their interpretation should be more age-aware and context-sensitive across heterogeneous patient groups, as CRP values in real-world practice are typically interpreted alongside age and other clinical features rather than stand-alone thresholds. Importantly, our analytical framework was designed to identify adjusted group-level distributional patterns in CRP rather than patient-level diagnostic thresholds or subtle clinically actionable differences in individual cases. Therefore, this study was not designed to determine the minimum detectable or clinically actionable subtle differences in CRP, and the observed group-level associations should not be used for individual patient management without additional clinical context. In adults, the use of fixed CRP thresholds may increase the risk of overinterpreting moderate CRP elevations as indicating bacterial infection or greater disease severity, particularly when age-related clinical background is not taken into account. This interpretation is supported by evidence from Danish general practice, where the CRP level associated with antibiotic prescribing varied according to age and presenting signs and symptoms rather than remaining constant across patients [24]. Age-aware interpretation of CRP may therefore be important to reduce potential diagnostic misclassification in adult patients presenting with respiratory symptoms.

In addition to the age effect, the multivariable analysis showed that virus-specific CRP associations were selective rather than universal. As multiple-virus detections are common in real-world multiplex respiratory PCR testing, particularly in pediatric settings, coinfected episodes were retained in the analytic framework and statistically accounted for by adjusting for both the total number of detected viruses and virus-specific indicators. However, this approach cannot fully determine which detected virus was the primary driver of a given clinical episode. Adenovirus remained independently associated with higher CRP levels after adjustment, confirming that this virus can elicit a comparatively strong inflammatory response [25,26]. In contrast, influenza A/B, RSV A/B, and parainfluenza virus types 1 and 3 were independently associated with relatively lower adjusted CRP levels within the multivariable framework. 

These inverse associations should not be interpreted as evidence that influenza A/B, RSV A/B, or parainfluenza viruses actively suppress CRP or reduce CRP below a healthy or pre-infection baseline. Rather, they indicate that, among clinically tested episodes with the same modeled covariate structure, these virus-positive episodes had relatively lower adjusted CRP levels than episodes with viruses showing stronger inflammatory profiles, particularly adenovirus, or than the broader virus-positive clinical background represented in the model. This interpretation is not inconsistent with previous studies showing that respiratory virus infections can produce mild CRP elevation, as the present analysis estimated relative differences across heterogeneous clinical episodes rather than absolute within-patient changes from a pre-infection baseline. 

hMPV, rhinovirus, enterovirus, and bocavirus did not show significant independent associations after adjustment, suggesting that crude differences observed in descriptive analyses may partly reflect age composition or other case-mix factors rather than a consistent virus-specific effect. However, direct microbiological data on bacterial co-infection were not linked in this dataset. Therefore, these virus-associated differences should not be interpreted as evidence that individual viruses intrinsically produced stronger or weaker inflammatory responses independent of possible concurrent bacterial infection.

Importantly, this interpretation was preserved in an additional analysis restricted to strict single-virus-positive episodes, which was conducted to reduce attributional ambiguity in episodes with multiple viral detections. In that restricted model, age remained the dominant correlate of CRP, whereas virus-specific associations remained selective, with a positive association observed for adenovirus, and negative associations observed for influenza B, RSV A/B, and parainfluenza virus types 1 and 3. These findings further support the view that the age-structured host-related clinical background contributes more strongly to CRP variation than viral identity alone, even when the analysis is limited to single-virus detections.

The high-CRP exclusion sensitivity analysis provided further support for this interpretation. After excluding episodes with CRP concentrations > 20 mg/dL, the magnitude of the adult and older-adult age effects was attenuated, as expected, but the age gradient remained the dominant feature of the model. Adenovirus also retained a positive association, and several inverse virus-specific associations, particularly for influenza B, RSV A/B, and parainfluenza virus type 1, persisted. These findings indicate that the principal conclusions were not driven solely by a small number of episodes with extreme inflammatory responses, although some virus-specific estimates were attenuated after excluding very high CRP values. In this restricted analysis, the total number of positive viral targets showed a modest negative association with CRP; however, this finding did not alter the dominant age-related gradient or the main virus-specific interpretation.

Clinically, these findings indicate that CRP should not be interpreted as a uniform marker of “viral severity”. A moderately elevated CRP result does not necessarily imply bacterial co-infection, and a relatively low CRP result does not exclude clinically relevant viral infection [27]. Additionally, extremely high CRP values should be interpreted with caution, as they may reflect bacterial superinfection, clinical complications, or greater overall illness severity rather than virus-specific effects alone. Bacterial co-infection was not directly assessed in this dataset; therefore, CRP differences across virus-associated groups should be interpreted cautiously in both pediatric and adult patients. Other host-response biomarkers, such as myxovirus-resistance protein A, may complement CRP in differentiating viral from bacterial respiratory infections [28]. Instead, CRP values may depend on both the patient’s age-related clinical context and the broader clinical setting in which viral detection occurs.

Although both host and pathogen factors contributed to CRP variation, their contributions were clearly not equivalent. The age gradient was substantially larger than the effect of any individual virus, whereas the male sex effect was statistically significant but modest. Neither the total number of detected viruses nor the calendar month showed a strong independent association in the adjusted model. Collectively, these findings support the interpretation that CRP during respiratory virus testing primarily reflects an age-structured host-related clinical context along with selected pathogen-specific effects. Additionally, this host-related context likely reflects biological aging, as well as age-related differences in comorbidity burden and tertiary-care case mix. These overall patterns were broadly consistent in sensitivity analyses restricted to the first eligible episode per patient, supporting the reliability of the main findings against potential bias from repeated testing.

Despite the widespread adoption of multiplex respiratory PCR testing, viral identification alone has not consistently reduced antibiotic prescribing in real-world practice [29,30]. Our findings suggest that one reason may be that inflammatory markers are often interpreted without sufficient age-related or pathogen-related context. An integrated diagnostic stewardship framework combining PCR results with age-aware interpretation of CRP may therefore provide more clinically interpretable context than either approach alone. In older patients, recognition of a higher age-dependent background CRP distribution observed among virus-not-detected-by-PCR episodes may aid in avoiding overinterpretation of moderate elevations. In younger patients, awareness that certain viruses, such as adenovirus, may be associated with comparatively higher CRP levels can support more cautious interpretation of inflammatory responses in the absence of other evidence for bacterial infection.

This study has some limitations. First, as a retrospective study conducted at a single tertiary-care hospital, the findings may reflect institutional case mix, referral patterns, and testing thresholds and may not be fully generalizable to primary care, outpatient, or community-based settings. Additionally, respiratory virus detection was performed using two different multiplex PCR platforms across the 2008–2024 study period. Although the analysis was restricted to viral targets consistently included across both platforms, and calendar year was included in the adjusted models, residual confounding related to inter-platform differences, changes in testing indications, and shifts in patient case mix cannot be fully excluded. Nevertheless, the additional sensitivity analysis restricted to the later real-time RT-PCR platform period from 2013 to 2024 showed that the dominant age-related CRP gradient and selective virus-specific associations were broadly preserved, supporting the robustness of the main interpretation while not eliminating all platform-related concerns. 

Second, detailed information on bacterial co-infection, antibiotic exposure, specific comorbidities (such as chronic obstructive pulmonary disease), autoimmune disease, malignancies, immunosuppressive status, symptom duration, and clinical severity was not uniformly available. This is particularly important because CRP is a time-dependent inflammatory marker, and measurements obtained early versus late in the illness course may yield different virus–CRP associations. Moreover, CRP was measured at a single clinical time point rather than serially; therefore, CRP kinetics and within-patient inflammatory trajectories could not be evaluated, limiting causal inference regarding virus-specific inflammatory responses. This is an important interpretive limitation because these factors may substantially influence CRP levels in pediatric and adult patients, particularly when CRP values are markedly elevated. Although the high-CRP exclusion sensitivity analysis showed broadly similar patterns, residual confounding by bacterial co-infection, clinical severity, comorbid inflammatory conditions, or treatment status could not be fully eliminated. Therefore, distinguishing between virus-associated CRP patterns and broader host-related clinical conditions or superimposed bacterial and mixed infectious processes is difficult. Furthermore, the prevalence of these unmeasured confounders may have differed across age groups; such age-dependent variations in bacterial co-infection, comorbidity burden, immunosuppressive status, and illness severity could have contributed to the observed age–CRP gradient. Moreover, because no direct inflammatory mediators, such as cytokines, were measured, the term ‘clinical and epidemiological context based on CRP patterns’ in this study should be interpreted as an epidemiological and clinical framework based on CRP patterns rather than as a direct characterization of specific immune pathways. 

Third, although the primary analysis was performed at the test-episode level, residual within-patient correlation cannot be entirely excluded. However, similar directions and overall patterns were observed in the sensitivity analysis restricted to the first eligible episode per patient, suggesting that the principal findings were less likely to be explained solely by repeated testing. 

Fourth, some virus–age strata, particularly among adolescents and older adults, for less frequently detected viruses, included small numbers of observations; therefore, subgroup-specific descriptive estimates should be interpreted cautiously. 

Fifth, CRP is a nonspecific inflammatory biomarker, and this study evaluated association rather than causation. Additional integration with other host-response markers, such as procalcitonin or cytokine profiles, may further improve diagnostic interpretation. Additionally, virus-not-detected-by-PCR episodes should not be regarded as true healthy controls, because this category may include bacterial infections, non-infectious inflammatory conditions, pathogens not covered by the PCR panel, and false-negative results related to specimen timing or test performance.

Future studies should prospectively evaluate age- and context-aware CRP interpretation strategies and assess their impact on antibiotic prescribing and clinical outcomes [31]. Serial CRP measurements and kinetics were previously suggested to offer additional diagnostic value beyond a single absolute CRP value [32,33]. Additionally, longitudinal studies incorporating serial CRP measurements and rates of CRP change from early infection through recovery may aid in distinguishing between acute infection-related CRP changes and age- or comorbidity-related clinical background across age groups.

## 5. Conclusions

The findings suggest that the CRP differences observed in respiratory virus testing were more closely associated with an age-structured host-related clinical background than the individual viruses alone. CRP markedly increased with advancing age in virus-positive and virus-not-detected-by-PCR groups. After multivariable adjustment, selective relative differences in CRP were observed across virus-positive episodes, supporting cautious interpretation of these findings as model-based associations rather than definitive virus-specific effects on CRP production. However, these associations should be interpreted cautiously in the absence of direct data on bacterial co-infection, comorbidities, and other clinical contributors to CRP elevation. Established CRP cut-offs remain clinically useful in routine practice; however, their interpretation should be age-aware and context-sensitive, particularly in heterogeneous patients undergoing respiratory virus testing. The virus-not-detected-by-PCR group should be interpreted as a clinically heterogeneous background group rather than a healthy control group, because it may include infections due to pathogens not covered by the PCR panel, false-negative PCR results, or non-infectious inflammatory conditions. Accordingly, CRP should be interpreted as a non-specific and composite host-response indicator whose clinical meaning depends on age, pathogen context, and other unmeasured contributors.

## Figures and Tables

**Table 1:** Virus detection status and age-stratified serum median CRP concentrations.

Virus	<1 Year, Median [IQR] (mg/dL)	1–12 Years, Median [IQR] (mg/dL)	13–18 Years, Median [IQR] (mg/dL)	19–64 Years, Median [IQR] (mg/dL)	≥65 Years, Median [IQR] (mg/dL)	*p*-Value
Adenovirus	0.63 [0.22–2.09] (*n* = 305)	1.54 [0.56–3.84] (*n* = 1416)	0.85 [0.34–2.03] (*n* = 24)	4.42 [0.58–9.30] (*n* = 52)	5.00 [1.13–14.80] (*n* = 37)	<0.001
Bocavirus	0.34 [0.10–0.86] (*n* = 103)	0.76 [0.27–2.39] (*n* = 289)	−(*n* = 0)	3.93 [2.77–6.28] (*n* = 6)	17.49 [14.59–19.30] (*n* = 4)	<0.001 ^†^
Enterovirus	0.41 [0.16–1.40] (*n* = 71)	0.58 [0.31–1.90] (*n* = 172)	0.99 [0.88–2.47] (*n* = 3)	3.11 [0.49–11.30] (*n* = 12)	7.62 [3.81–12.20] (*n* = 21)	<0.001 ^†^
Influenza A	0.30 [0.08–0.99] (*n* = 175)	0.78 [0.26–2.38] (*n* = 380)	1.21 [0.40–3.68] (*n* = 27)	3.26 [1.10–9.57] (*n* = 178)	6.17 [2.17–13.10] (*n* = 202)	<0.001
Influenza B	0.32 [0.10–0.83] (*n* = 30)	0.51 [0.20–1.48] (*n* = 188)	0.32 [0.24–0.46] (*n* = 18)	3.31 [0.56–9.51] (*n* = 43)	5.58 [3.01–8.52] (*n* = 18)	<0.001
Parainfluenza virus 1	0.33 [0.09–0.84] (*n* = 128)	0.64 [0.15–1.82] (*n* = 228)	11.50 [7.23–15.80] (*n* = 2)	11.21 [4.70–19.10] (*n* = 9)	9.07 [2.28–26.70] (*n* = 16)	<0.001 ^†^
Parainfluenza virus 2	0.42 [0.05–0.61] (*n* = 33)	0.51 [0.20–1.60] (*n* = 87)	3.17 [1.64–4.70] (*n* = 2)	0.68 [0.64–0.71] (*n* = 2)	12.49 [5.88–14.20] (*n* = 6)	<0.001 ^†^
Parainfluenza virus 3	0.36 [0.10–1.18] (*n* = 348)	0.66 [0.21–2.04] (*n* = 444)	0.52 [0.24–0.91] (*n* = 16)	4.03 [1.77–7.86] (*n* = 35)	4.63 [1.35–11.00] (*n* = 61)	<0.001
RSV A	0.25 [0.05–0.82] (*n* = 896)	0.58 [0.23–1.68] (*n* = 510)	5.92 [4.20–8.75] (*n* = 6)	1.14 [0.45–5.03] (*n* = 16)	8.94 [4.65–19.80] (*n* = 32)	<0.001 ^†^
RSV B	0.32 [0.05–0.91] (*n* = 668)	0.67 [0.24–2.04] (*n* = 494)	1.01 [0.40–6.21] (*n* = 5)	4.04 [0.78–11.00] (*n* = 41)	4.58 [2.08–11.90] (*n* = 50)	<0.001 ^†^
Rhinovirus	0.46 [0.14–1.34] (*n* = 1233)	0.86 [0.31–2.53] (*n* = 2048)	1.23 [0.44–3.98] (*n* = 88)	2.22 [0.75–7.57] (*n* = 89)	7.26 [3.35–13.80] (*n* = 101)	<0.001
hMPV	0.46 [0.19–1.35] (*n* = 243)	0.94 [0.33–2.61] (*n* = 432)	1.31 [1.07–2.04] (*n* = 9)	3.81 [1.18–9.32] (*n* = 47)	5.32 [2.31–11.70] (*n* = 71)	<0.001 ^†^

Values are presented as untransformed median [IQR] (mg/dL), with the number of virus-positive observations in parentheses. *p*-values indicate within-virus comparisons of CRP distributions across age groups using the Kruskal–Wallis test. ^†^ For viruses with one or more age–virus cells containing fewer than 10 observations, *p*-values were retained for transparency but should be interpreted as descriptive only because subgroup medians may be unstable and should not be regarded as robust inferential comparisons. CRP, C-reactive protein; IQR, interquartile range; RSV, respiratory syncytial virus; hMPV, human metapneumovirus.

**Table 2:** C-reactive protein concentrations among virus-not-detected-by-PCR episodes according to age group.

Age Group (Years)	Tests, *n*	Median CRP (mg/dL)	IQR (mg/dL)	*p*-Value
<1	1829	0.38	0.09–1.62	<0.001
1–12	2198	0.95	0.31–2.88	
13–18	347	1.36	0.33–5.33	
19–64	1753	4.71	1.42–10.96	
≥65	2618	7.42	2.87–13.83	

CRP concentrations are presented as median and interquartile range (IQR). CRP, C-reactive protein.

## Data Availability

The data supporting the findings of this study were derived from clinical records at Dankook University Hospital and are subject to ethical and legal restrictions. To safeguard patient privacy and confidentiality, the raw datasets cannot be made publicly available. However, de-identified aggregated data may be obtained from the corresponding author on reasonable request, subject to approval by the Institutional Review Board.

## Supplementary Materials

The following supporting information can be downloaded at: https://www.mdpi.com/article/10.3390/pathogens15060583/s1, Table S1: Multivariable linear regression of log-transformed CRP levels adjusted for age group, sex, calendar month, calendar year, total number of positive viral targets and virus type; Table S2: Sensitivity analysis of multivariable linear regression of log-transformed CRP: virus-specific associations and selected covariates; Table S3: Additional multivariable linear regression analysis of log-transformed CRP levels in the strict single-virus-positive subset; Table S4: Sensitivity analysis excluding episodes with very high CRP concentrations (>20 mg/dL); Table S5: Number of observations in each virus–age stratum used for Table 1 and Figure 1; Table S6: Sensitivity analysis restricted to the later real-time RT-PCR platform period, 2013–2024.

## Abbreviations

The following abbreviations are used in this manuscript:

CRP	C-reactive protein
hMPV	Human metapneumovirus
IQR	Interquartile range
LIS	Laboratory information system
RSV	Respiratory syncytial virus
