# Supplementary material for: Age-Structured Clinical Background Is More Strongly Associated with C-Reactive Protein Levels than Individual Respiratory Viruses During Respiratory Virus Testing
Source: Pathogens. 2026 May 28;15(6):583. doi: 10.3390/pathogens15060583 (PMC13305711; doi:10.3390/pathogens15060583)
Supplement: Supplementary file 1 [file pathogens-15-00583-s001.zip › pathogens-4247659-supplementary.pdf]

Supplementary Materials

Table S1. Multivariable linear regression of log-transformed CRP levels adjusted for age group, sex, calendar month, calendar year, total number of positive viral targets, and virus type.

Term	Estimate (β)	SE	t value	*p*-value	% change	95% CI for β	95% CI for % change
(Intercept)	0.6056	0.0465	13.019	<0.001	83.2	0.5144 to 0.6967	67.3 to 100.7
Age group							
1–12 years vs. <1 year	0.2486	0.0140	17.725	<0.001	28.2	0.2211 to 0.2762	24.7 to 31.8
13–18 years vs. <1 year	0.4511	0.0343	13.135	<0.001	57.0	0.3838 to 0.5185	46.8 to 67.9
19–64 years vs. <1 year	1.0637	0.0205	51.777	<0.001	189.7	1.0234 to 1.1039	178.2 to 201.6
≥65 years vs. <1 year	1.3660	0.0203	67.233	<0.001	291.9	1.3262 to 1.4058	276.7 to 307.8
Sex							
Male vs. Female	0.0611	0.0112	5.446	<0.001	6.3	0.0391 to 0.0831	4.0 to 8.7
Other covariate							
Total number of positive viral targets	−0.0393	0.0268	−1.467	0.142	−3.9	−0.0918 to 0.0132	−8.8 to 1.3
Calendar month							
February vs. January	−0.0124	0.0276	−0.448	0.654	−1.2	−0.0665 to 0.0417	−6.4 to 4.3
March vs. January	−0.0383	0.0272	−1.407	0.160	−3.8	−0.0917 to 0.0151	−8.8 to 1.5
April vs. January	−0.0057	0.0268	−0.212	0.832	−0.6	−0.0583 to 0.0469	−5.7 to 4.8
May vs. January	−0.0009	0.0267	−0.034	0.973	−0.1	−0.0533 to 0.0515	−5.2 to 5.3
June vs. January	−0.0299	0.0285	−1.050	0.294	−2.9	−0.0857 to 0.0259	−8.2 to 2.6
July vs. January	−0.0583	0.0291	−2.002	0.045	−5.7	−0.1155 to −0.0012	−10.9 to −0.1
August vs. January	0.0229	0.0289	0.791	0.429	2.3	−0.0338 to 0.0796	−3.3 to 8.3
September vs. January	0.0037	0.0293	0.125	0.901	0.4	−0.0537 to 0.0611	−5.2 to 6.3
October vs. January	−0.0017	0.0277	−0.063	0.950	−0.2	−0.0559 to 0.0525	−5.4 to 5.4
November vs. January	0.0129	0.0260	0.497	0.619	1.3	−0.0380 to 0.0638	−3.7 to 6.6
December vs. January	0.0283	0.0253	1.117	0.264	2.9	−0.0213 to 0.0779	−2.1 to 8.1
Calendar year							
2009 vs. 2008	0.0825	0.0454	1.816	0.069	8.6	−0.0066 to 0.1720	−0.7 to 18.7
2010 vs. 2008	0.0195	0.0436	0.447	0.655	2.0	−0.0659 to 0.1050	−6.4 to 11.1
2011 vs. 2008	0.0185	0.0440	0.421	0.674	1.9	−0.0677 to 0.1050	−6.6 to 11.0
2012 vs. 2008	−0.0098	0.0449	−0.218	0.828	−1.0	−0.0979 to 0.0783	−9.3 to 8.1
2013 vs. 2008	−0.0194	0.0444	−0.437	0.662	−1.9	−0.1060 to 0.0676	−10.1 to 7.0
2014 vs. 2008	−0.0263	0.0445	−0.590	0.555	−2.6	−0.1130 to 0.0609	−10.7 to 6.3
2015 vs. 2008	−0.0171	0.0449	−0.380	0.704	−1.7	−0.1050 to 0.0709	−10.0 to 7.4
2016 vs. 2008	0.0094	0.0441	0.214	0.831	0.9	−0.0770 to 0.0959	−7.4 to 10.1

Term	Estimate (β)	SE	t value	*p*-value	% change	95% CI for β	95% CI for % change
2017 vs. 2008	−0.0734	0.0452	−1.622	0.105	−7.1	−0.1620 to 0.0153	−15.0 to 1.5
2018 vs. 2008	−0.0449	0.0447	−1.005	0.315	−4.4	−0.1320 to 0.0427	−12.4 to 4.4
2019 vs. 2008	−0.0870	0.0449	−1.936	0.053	−8.3	−0.1750 to 0.0011	−16.1 to 0.1
2020 vs. 2008	−0.0547	0.0490	−1.117	0.264	−5.3	−0.1510 to 0.0413	−14.0 to 4.2
2021 vs. 2008	−0.0384	0.0509	−0.755	0.450	−3.8	−0.1380 to 0.0613	−12.9 to 6.3
2022 vs. 2008	−0.0954	0.0486	−1.961	0.050	−9.1	−0.1910 to −0.0001	−17.4 to 0.0
2023 vs. 2008	0.0375	0.0485	0.773	0.440	3.8	−0.0576 to 0.1330	−5.6 to 14.2
2024 vs. 2008	0.1984	0.0568	3.493	<0.001	21.9	0.0871 to 0.3100	9.1 to 36.3
Virus type							
Influenza A	−0.0795	0.0369	−2.154	0.031	−7.6	−0.1518 to −0.0071	−14.1 to −0.7
Influenza B	−0.1783	0.0524	−3.404	<0.001	−16.3	−0.2811 to −0.0754	−24.5 to −7.3
RSV A	−0.1788	0.0345	−5.181	<0.001	−16.4	−0.2464 to −0.1112	−21.8 to −10.5
RSV B	−0.1265	0.0351	−3.605	<0.001	−11.9	−0.1953 to −0.0577	−17.7 to −5.6
hMPV	−0.0055	0.0387	−0.143	0.887	−0.6	−0.0814 to 0.0704	−7.8 to 7.3
Parainfluenza 1	−0.1265	0.0474	−2.670	0.008	−11.9	−0.2193 to −0.0337	−19.7 to −3.3
Parainfluenza 2	−0.1391	0.0716	−1.942	0.052	−13.0	−0.2794 to 0.0013	−24.4 to 0.1
Parainfluenza 3	−0.0899	0.0380	−2.368	0.018	−8.6	−0.1644 to −0.0155	−15.2 to −1.5
Rhinovirus	0.0035	0.0304	0.114	0.909	0.3	−0.0561 to 0.0630	−5.5 to 6.5
Adenovirus	0.2431	0.0333	7.303	<0.001	27.5	0.1778 to 0.3083	19.5 to 36.1
Enterovirus	−0.0354	0.0542	−0.653	0.514	−3.5	−0.1417 to 0.0708	−13.2 to 7.3
Bocavirus	0.0144	0.0475	0.303	0.762	1.4	−0.0787 to 0.1075	−7.6 to 11.4

Estimates were obtained from a multivariable linear regression model with log(CRP + 1) as the dependent variable. Percent changes were calculated as (exp[β] − 1) × 100 and should be interpreted as approximate back-transformed relative differences from the log(CRP + 1) model rather than exact proportional changes in raw CRP concentrations. The model simultaneously included age group, sex, calendar month, calendar year, total number of positive viral targets, and 12 virus-specific indicator variables. The reference categories were age <1 year for age-group comparisons, female sex for sex comparisons, January for calendar month, and 2008 for calendar year. Age group and sex were entered as separate categorical predictors without an interaction term. CI, confidence interval; SE, standard error; CRP, C-reactive protein; hMPV, human metapneumovirus; RSV, respiratory syncytial virus

Table S2. Sensitivity analysis of multivariable linear regression of log-transformed CRP: virus-specific associations and selected covariates.

Variable	% change (95% CI)	*p*-value
Influenza A	-9.9% (-16.9 to -2.3)	0.012
Influenza B	-13.9% (-23.3 to -3.4)	0.011
RSV A	-17.9% (-24.0 to -11.5)	<0.001
RSV B	-12.9% (-19.4 to -5.9)	<0.001
hMPV	-1.0% (-9.2 to 7.9)	0.815
Parainfluenza 1	-12.0% (-20.9 to -2.2)	0.018
Parainfluenza 2	-13.9% (-26.7 to 1.2)	0.069
Parainfluenza 3	-10.2% (-17.5 to -2.3)	0.012
Rhinovirus	-0.7% (-7.2 to 6.3)	0.838
Adenovirus	25.9% (16.9 to 35.6)	<0.001
Enterovirus	-5.5% (-16.3 to 6.8)	0.367
Bocavirus	-0.8% (-11.1 to 10.7)	0.888
Selected covariates		
Age 1–12 years vs. <1 year	30.8% (26.8 to 34.9)	<0.001
Age 13–18 years vs. <1 year	54.4% (43.2 to 66.4)	<0.001
Age 19–64 years vs. <1 year	191.5% (179.2 to 204.4)	<0.001
Age ≥65 years vs. <1 year	296.3% (279.7 to 313.9)	<0.001
Male vs. Female	8.2% (5.6 to 10.9)	<0.001
Positive count	-2.8% (-8.4 to 3.2)	0.360

Estimates were obtained from a sensitivity analysis restricted to the first eligible episode per patient to assess the influence of repeated testing within individuals. CI, confidence interval; CRP, C-reactive protein; hMPV, human metapneumovirus; RSV, respiratory syncytial virus

Table S3. Additional multivariable linear regression analysis of log-transformed CRP levels in the strict single-virus-positive subset.

Variable	Estimate (β)	SE	95% CI	Adjusted % change	*p*-value
Age 1–12 vs. <1	0.2468	0.0183	0.2110 to 0.2826	28.0	<0.001
Age 13–18 vs. <1	0.3917	0.0544	0.2850 to 0.4980	48.0	<0.001
Age 19–64 vs. <1	1.0300	0.0371	0.9570 to 1.1030	180.0	<0.001
Age ≥65 vs. <1	1.3573	0.0373	1.2840 to 1.4300	289.0	<0.001
Male vs. female	-0.0127	0.0159	-0.0439 to 0.0185	-1.3	0.425
Influenza A	-0.0920	0.0696	-0.2280 to 0.0444	-8.8	0.186
Influenza B	-0.2039	0.0780	-0.3570 to -0.0510	-18.4	0.009
RSV A	-0.2116	0.0680	-0.3450 to -0.0782	-19.1	0.002
RSV B	-0.1689	0.0684	-0.3030 to -0.0349	-15.5	0.014
hMPV	-0.0219	0.0692	-0.1580 to 0.1140	-2.2	0.752
Parainfluenza virus 1	-0.1925	0.0761	-0.3420 to -0.0433	-17.5	0.011
Parainfluenza virus 2	-0.1409	0.1015	-0.3400 to 0.0580	-13.1	0.165
Parainfluenza virus 3	-0.1634	0.0685	-0.2980 to -0.0291	-15.1	0.017
Rhinovirus	-0.0139	0.0642	-0.1400 to 0.1120	-1.4	0.828
Adenovirus	0.2707	0.0675	0.1380 to 0.4030	31.1	<0.001
Enterovirus	0.1115	0.1032	-0.0908 to 0.3140	11.8	0.280

Bocavirus was used as the omitted reference category in the strict single-virus-positive model because each episode contained exactly one detected viral target. CI, confidence interval; CRP, C-reactive protein; hMPV, human metapneumovirus; RSV, respiratory syncytial virus

Table S4. Sensitivity analysis excluding episodes with very high CRP concentrations (>20 mg/dL).

Group	Variable	Estimate (β)	SE	95% CI for β	Adjusted % change (95% CI)	*p*-value
Selected covariates	Age 1–12 years vs. <1 year	0.2424	0.0131	0.2167 to 0.2682	27.4% (24.2 to 30.8)	<0.001
Selected covariates	Age 13–18 years vs. <1 year	0.4045	0.0324	0.3410 to 0.4681	49.8% (40.6 to 59.7)	<0.001
Selected covariates	Age 19–64 years vs. <1 year	0.9044	0.0198	0.8656 to 0.9433	147.0% (137.7 to 156.8)	<0.001
Selected covariates	Age ≥65 years vs. <1 year	1.2061	0.0197	1.1675 to 1.2447	234.0% (221.4 to 247.2)	<0.001
Selected covariates	Male vs. Female	0.0513	0.0107	0.0305 to 0.0722	5.3% (3.1 to 7.5)	<0.001
Selected covariates	Total number of positive viral targets	−0.0660	0.0254	−0.1159 to −0.0161	−6.4% (−10.9 to −1.6)	0.009
Virus-specific associations	Influenza A	−0.0488	0.0351	−0.1175 to 0.0200	−4.8% (−11.1 to 2.0)	0.165
Virus-specific associations	Influenza B	−0.1537	0.0494	−0.2506 to −0.0568	−14.2% (−22.2 to −5.5)	0.002
Virus-specific associations	RSV A	−0.1513	0.0326	−0.2152 to −0.0873	−14.0% (−19.4 to −8.4)	<0.001
Virus-specific associations	RSV B	−0.0985	0.0332	−0.1635 to −0.0334	−9.4% (−15.1 to −3.3)	0.003
Virus-specific associations	hMPV	0.0302	0.0366	−0.0415 to 0.1020	3.1% (−4.1 to 10.7)	0.409
Virus-specific associations	Parainfluenza virus 1	−0.1192	0.0448	−0.2071 to −0.0313	−11.2% (−18.7 to −3.1)	0.008
Virus-specific associations	Parainfluenza virus 2	−0.1067	0.0673	−0.2386 to 0.0251	−10.1% (−21.2 to 2.5)	0.113
Virus-specific associations	Parainfluenza virus 3	−0.0527	0.0359	−0.1230 to 0.0177	−5.1% (−11.6 to 1.8)	0.142
Virus-specific associations	Rhinovirus	0.0298	0.0288	−0.0267 to 0.0862	3.0% (−2.6 to 9.0)	0.301
Virus-specific associations	Adenovirus	0.2679	0.0315	0.2061 to 0.3297	30.7% (22.9 to 39.1)	<0.001
Virus-specific associations	Enterovirus	−0.0077	0.0512	−0.1081 to 0.0927	−0.8% (−10.2 to 9.7)	0.880
Virus-specific associations	Bocavirus	0.0397	0.0447	−0.0480 to 0.1274	4.0% (−4.7 to 13.6)	0.375

Estimates were obtained from a multivariable linear regression model with log(CRP + 1) as the dependent variable after excluding episodes with CRP concentrations >20 mg/dL. A total of 646 episodes (3.4%) were excluded, leaving 18,356 episodes in the restricted dataset. Percent changes were calculated as (exp[β] − 1) × 100 and should be interpreted as approximate back-transformed relative differences from the log(CRP + 1) model rather than exact proportional changes in raw CRP concentrations. The model included age group, sex, calendar month, calendar year, total number of positive viral targets, and virus-specific indicator variables. CI, confidence interval; CRP, C-reactive protein; hMPV, human metapneumovirus; RSV, respiratory syncytial virus.

Table S5. Number of observations in each virus–age stratum used for Table 1 and Figure 1.

Virus	<1 year	1–12 years	13–18 years	19–64 years	≥65 years
Adenovirus	305	1416	24	52	37
Bocavirus	103	289	0	6	4
Enterovirus	71	172	3	12	21
Influenza A	175	380	27	178	202
Influenza B	30	188	18	43	18
Parainfluenza virus 1	128	228	2	9	16
Parainfluenza virus 2	33	87	2	2	6
Parainfluenza virus 3	348	444	16	35	61
RSV A	896	510	6	16	32
RSV B	668	494	5	41	50
Rhinovirus	1233	2048	88	89	101
hMPV	243	432	9	47	71

Values represent the number of virus-positive observations contributing to each virus–age cell. Cells with fewer than 10 observations should be interpreted cautiously because median CRP estimates may be unstable. CRP, C-reactive protein; hMPV, human metapneumovirus; RSV, respiratory syncytial virus.

Table S6. Sensitivity analysis restricted to the later real-time RT-PCR platform period, 2013–2024.

**Variable**	**Estimate (β)**	**SE**	**95% CI**	**Adjusted % change (95% CI)**	**p-value**
Age 1–12 years vs. <1 year	0.2264	0.0177	0.1917 to 0.2612	25.4% (21.1 to 29.9)	<0.001
Age 13–18 years vs. <1 year	0.4265	0.0396	0.3488 to 0.5041	53.2% (41.7 to 65.6)	<0.001
Age 19–64 years vs. <1 year	1.0306	0.0245	0.9825 to 1.0786	180.3% (167.1 to 194.1)	<0.001
Age ≥65 years vs. <1 year	1.3387	0.0249	1.2899 to 1.3876	281.4% (263.3 to 300.5)	<0.001
Male vs. female	0.0740	0.0137	0.0471 to 0.1009	7.7% (4.8 to 10.6)	<0.001
Total number of positive viral targets	−0.0423	0.0326	−0.1062 to 0.0216	−4.1% (−10.1 to 2.2)	0.195
Influenza A	−0.1210	0.0465	−0.2121 to −0.0298	−11.4% (−19.1 to −2.9)	0.009
Influenza B	−0.2657	0.0654	−0.3939 to −0.1376	−23.3% (−32.6 to −12.9)	<0.001
RSV A	−0.2021	0.0454	−0.2910 to −0.1132	−18.3% (−25.2 to −10.7)	<0.001
RSV B	−0.1483	0.0444	−0.2353 to −0.0612	−13.8% (−21.0 to −5.9)	<0.001
hMPV	−0.0593	0.0484	−0.1542 to 0.0357	−5.8% (−14.3 to 3.6)	0.221
Parainfluenza virus 1	−0.1391	0.0644	−0.2654 to −0.0129	−13.0% (−23.3 to −1.3)	0.031
Parainfluenza virus 2	−0.1120	0.0879	−0.2843 to 0.0603	−10.6% (−24.7 to 6.2)	0.203
Parainfluenza virus 3	−0.1045	0.0467	−0.1961 to −0.0129	−9.9% (−17.8 to −1.3)	0.025
Rhinovirus	0.0029	0.0369	−0.0694 to 0.0751	0.3% (−6.7 to 7.8)	0.938
Adenovirus	0.2316	0.0415	0.1502 to 0.3129	26.1% (16.2 to 36.7)	<0.001
Enterovirus	−0.0332	0.0581	−0.1471 to 0.0807	−3.3% (−13.7 to 8.4)	0.568
Bocavirus	0.0223	0.0516	−0.0790 to 0.1235	2.3% (−7.6 to 13.1)	0.666
